# Fill factor in organic solar cells can exceed the Shockley-Queisser limit

**DOI:** 10.1038/srep11478

**Published:** 2015-06-22

**Authors:** Vasily A. Trukhanov, Vladimir V. Bruevich, Dmitry Yu. Paraschuk

**Affiliations:** 1 Faculty of Physics and International Laser Center, M.V. Lomonosov Moscow State University, Moscow, 119991, Russia

## Abstract

The ultimate efficiency of organic solar cells (OSC) is under active debate. The solar cell efficiency is calculated from the current-voltage characteristic as a product of the open-circuit voltage (*V*_*OC*_), short-circuit current (*J*_*SC*_), and the fill factor (*FF*). While the factors limiting *V*_*OC*_ and *J*_*SC*_ for OSC were extensively studied, the ultimate *FF* for OSC is scarcely explored. Using numerical drift-diffusion modeling, we have found that the *FF* in OSC can exceed the Shockley-Queisser limit (SQL) established for inorganic *p–n* junction solar cells. Comparing charge generation and recombination in organic donor-acceptor bilayer heterojunction and inorganic *p–n* junction, we show that such distinctive properties of OSC as interface charge generation and heterojunction facilitate high *FF*, but the necessary condition for *FF* exceeding the SQL in OSC is field-dependence of charge recombination at the donor-acceptor interface. These findings can serve as a guideline for further improvement of OSC.

The efficiency of organic solar cells (OSC) has shown an impressive growth for the recent years^1^ and their ultimate efficiency is under intense discussion^2^,^3^,^4^,^5^,^6^, with the Shockley-Queisser (SQ) model proposed for inorganic solar cells (ISC) being a reference point^7^. The photoactive layer of OSC is based on type-II heterojunction formed by two organic semiconductors with different electron affinities and ionization potentials, one of them is an electron donor, and the other is an electron acceptor^8^. The maximal achieved efficiencies are comparable for two different architectures of heterojunction: over 10% for the bulk one^9^ and 8.4% for the planar one^10^. The heterojunction is needed for dissociation of excitons that are generated upon light absorption in organic semiconductors. Exciton dissociation into free charges needs an extra energy so that the ultimate OSC efficiency is generally suggested to be lower than the Shockley-Queisser limit (SQL). This extra energy eventually reduces the solar cell voltage, specifically the open circuit voltage (*V*_*OC*_)^2^,^11^. However the photocurrent, in particular the short-circuit current density (*J*_*SC*_) for OSC can potentially exceed *J*_*SC*_ for a single *p–n* junction ISC due to the effect of singlet exciton fission, when one photon can produce two pairs of electrons and holes^12^. The cell efficiency also determined by the shape of its current-voltage characteristic, and this shape can be characterized by the fill factor (*FF*) that is the ratio of the maximum output power to the product of short-circuit current and *V*_*OC*_. As a result, the cell efficiency is proportional to the product of *V*_*OC*_, *J*_*SC*_, and *FF.* The SQ model establishes the theoretical limit for *FF* as a function of *V*_*OC*_^7^,^13^. For OSC the limiting factors for *J*_*SC*_ and *V*_*OC*_ were extensively studied^14^,^15^,^16^,^17^,^18^, but the ultimate *FF* for organic solar cells has been discussed only in a few works. The *FF* is assumed to be lower than that for ISC because of increased charge recombination^19^.

As *FF* is one of the key parameters determining the solar cell efficiency, in this Report we address the issue of the ultimate *FF* in OSC. We use numerical drift-diffusion modeling, which is a well-established approach in OSC study^20^,^21^,^22^,^23^. Using the model for bilayer organic solar cell described in our previous work^24^, we show that the *FF* in planar heterojunction OSC can exceed the SQL and substantiate this result by contrasting charge generation and recombination in organic heterojunction and *p–n* junction solar cells. Our approach is following: we confront drift-diffusion models for organic planar heterojunction and inorganic *p–n* junction (homojunction). The homojunction model presents an ideal inorganic solar cell with bulk generation of charges and their radiative recombination as the only loss of them. The most essential differences between these models are the junction type (homo/heterojunction), the charge generation-recombination spatial distribution (bulk/interface), and the recombination dependence on the electric field (dependent/independent). The planar heterojunction model simulates a bilayer OSC^20^,^24^ in which generation of free charges occurs only at the heterojunction interface, and their recombination happens in the bulk according to the Langevin model^25^ and at the interface via binding of electrons and holes into a charge-transfer state^26^. In the ISC model, the *FF* exactly follows the SQ model, whereas, in the OSC model, it can be higher. To reveal the origin of high *FF*, we replace step by step the essential features of the OSC/ISC model by the corresponding features of the ISC/OSC model and observe how the *FF* varies relative to the SQL. For example, we replace the heterojunction by a homojunction in the OSC model and the homojunction by a heterojunction in the ISC model, then we replace the interface recombination by the bulk one, etc. Following this way, we have found that the field-dependent charge recombination is responsible for the *FF* exceeding the SQL: the higher the electric field at the heterojunction interface, the less the interface charge recombination, and the steeper the current-voltage characteristic at voltages near the *V*_*OC*_.

## Results

### Modeling

In this section, we briefly describe the models of solar cells used in this work.

### Current-voltage characteristics of ideal inorganic solar cell

The textbook model for ISC gives the following expression for their current density-voltage (*J–V)* characteristics:


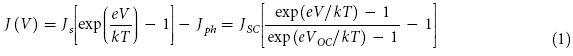


where *J*_*s*_ and *J*_*ph*_ are the saturation current and photocurrent densities, which can be uniquely expressed via the *J*_*SC*_ and *V*_*OC*_; *e* is the electron charge, *k* is the Boltzmann constant, *T* is the cell temperature. Equation (1) corresponds to an ideal solar cell with the zero series and infinite shunt resistances and with unity diode ideality factor.

### Model of bilayer organic solar cell

We use a numerical model recently developed for bilayer organic solar cells^24^ that accounts for generation, recombination, drift and diffusion of charge carriers, doping, and space charge effects in the active layers. Briefly, generation and recombination of free charges in OSC model occur via bound electron-hole (e/h) pairs at the donor-acceptor interface (charge transfer states) and depend on the interface electric field. In the bulk of the layers, the charges recombine according to the Langevin model. The input parameters of the model are the charge mobilities, band gap, layer thickness, doping level, and dielectric constant for each layer. As a reference, we use the input parameters for the most studied donor-acceptor material pair, poly-3-hexylthiophene and phenyl-C_61_-butyric acid methyl ester (P3HT/PCBM), which are given in Table 1. In addition, the active layers are equally doped by majority carriers (donor is *p*-doped, acceptor is *n*-doped) with concentration *N*_*d*_ = 10^24^ m^−3^, and the rate of bound e/h-pairs dissociation at zero field *k*_*diss*_(0) is set 100 times higher than the monomolecular recombination rate of bound e/h-pairs *k*. As a result, the modeled OSC have very high *FF*.

### Model of inorganic p–n junction solar cell

To reveal the origin of the high *FF*, we compare generation, transport, and recombination of charges in the bilayer OSC and *p*–*n* junction ISC. For this purpose, we developed a drift-diffusion *p–n* junction model for ISC similar to our model for bilayer OSC^24^. The details of the *p–n* junction model are given in Methods. The main difference between these models is that the generation of free charge carriers occurs in the bulk, i.e. in the whole volume of the active layer, and there is the only recombination channel via bulk bimolecular radiative recombination. As the input parameters, we use those typical to silicon *p–n* junction solar cells. Table 1 compares the features of OSC and ISC models.

### Organic solar cells with *FF* exceeding the Shockley-Queisser limit

**Figure 1:**
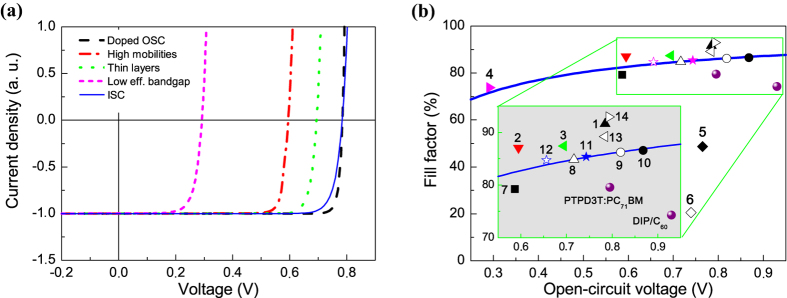
*J–V* characteristics and *FF* for high *FF* solar cells. (**a**) *J–V* characteristics for doped (the doping level is 10^24^ m^−3^, black dashed curve), high-mobility (mobilities of majority carriers *μ*_*n*_ = *μ*_*p*_ = 10^−5^ m^2^/(V s), red dash-dotted curve), thin (donor and acceptor layers thickness 30 nm instead of 50 nm for other OSC, green dotted curve), low-effective-bandgap OSC (acceptor LUMO and donor HOMO were symmetrically shifted so that their difference was lowered from 1.05 eV to 0.6 eV, magenta short-dashed curve) and for ideal inorganic *p–n* junction solar cell (blue solid curve). Other input parameters are given in Table 1 and are the same as for bilayer OSC, modeled in our previous work^24^. *J–V* characteristics for inorganic *p–n* junction solar cell (blue solid line) is plotted according to equation (1) with the same *J*_*SC*_ and *V*_*OC*_ as in the doped OSC, the temperature *T* is 300 K. (**b**) Shockley-Queisser limit for *FF* vs *V*_*OC*_ (solid line)^7^,^13^ and calculated *FF*s for various OSCs (points 1–14). Points (1–4) correspond to high-*FF* OSCs, their *J–V* curves are presented in Fig. 1a, other points (5–14) are our results which are described in the text: modeled OSC with bulk charge generation for different generation regions *Δx* = 1 nm and 10 nm (points 5 and 6 correspondingly), OSC with homojunction (point 7), OSC with constant interface recombination for different constant bound e/h-pair dissociation rates *k*_*diss*_ *=* *k*_*diss*_(0), *k*_*diss*_(*E*_*BI*_) and *k*_*diss*_(2*E*_*BI*_) (points 8, 9 and 10 correspondingly), ISC (point 11) and ISC with interface field dependent charge recombination similar to OSC (point 12), OSC with linear (point 13) and exponential-square-root (point 14) dependences of bound e/h-pair dissociation rate *k*_*diss*_(*E*_*i*_). The highest experimental FF for OSC (PTPD3T:PCBM polymer bulk heterojunction cell^27^ and DIP/C60 low-molecular bilayer solar cell^28^) are shown by spheres. The descriptions of each point are also given in Table 2.

The bilayer OSC model gives *J–V* characteristics with *FF* exceeding the SQL for a number of the input parameters sets. Figure 1a illustrates such four *J–V* characteristics (broken lines) for a doped, high-mobility, thin, and low-effective-bandgap OSC, which are described in details in the figure caption and in Table 2. The last three OSC are undoped. For comparison, *p–n* junction *J–V* curve calculated according to equation (1) (solid line) is plotted in Fig. 1a with the same *J*_*SC*_ and *V*_*OC*_ as for the doped OSC. As the ultimate *FF* of an inorganic *p–n* junction solar cell is a function of *V*_*OC*_ and does not depend on *J*_*SC*_^7^,^13^, one can use current-normalized *J–V* curves to compare the maximum possible *FF* in the OSC and ISC. In all the figures, the current density is normalized to the product *eG*_*S*_, where *G*_*S*_ is the interface generation rate of bound electron-hole pairs.

Figure 1b displays *FF* vs *V*_*OC*_ for various solar cells. The *FF*s of the doped (point 1), high-mobility (point 2), thin (point 3) and low-effective-bandgap OSC (point 4) are higher than that of the ideal inorganic *p–n* junction cell (solid line). The lower *V*_*OC*_ in the high-mobility OSC is due to the higher Langevin recombination rate within the layers. Although doping is not the necessary condition for *FF* exceeding the SQL, the most pronounced exceeding of the SQL by 7% is observed for the doped OSC (*FF* = 91.7%). Because of this, further we study the nature of high *FF* using mainly the doped bilayer OSC. Moreover, the latter can have higher performance than the undoped one: if the layers are doped by majority carriers (i.e., the donor layer is *p*-doped, and the acceptor layer is *n*-doped), all the photovoltaic parameters (*J*_*SC*_, *V*_*OC*_, *FF* and efficiency) can be increased with doping^24^. The reason is in a significant increase of the electric field at the donor-acceptor interface due to space charge caused by doping^24^. The other points in Fig. 1b (5–14) refer to our numerical experiments described below in the next sections. Table 2 summarizes all the data presented in Fig. 1b and compares the modeled *FF*s with the SQL for *FF*. The best experimental values for *FF* reported for bulk^27^ and planar^28^ heterojunction OSC are presented in Fig. 1b by spheres.

In the next sections, following our approach mentioned above, we study the origin of high *FF* in OSC.

### Generation of free charges: bulk vs interface

**Figure 2:**
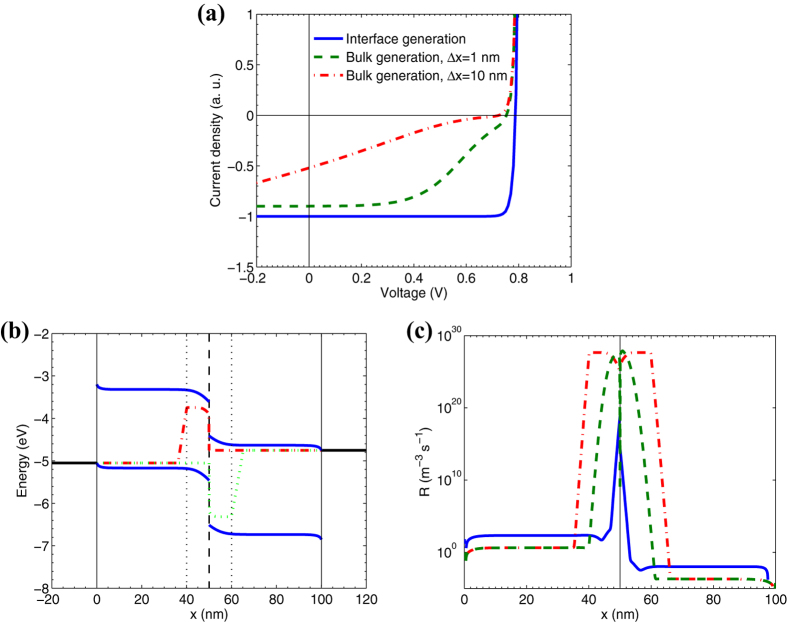
Bulk vs interface generation of free charges in OSC. (**a**) *J–V* characteristics for a OSC with interface (solid line) and bulk generation of free charges for two different widths of the generation region *Δx* = 1 nm (dashed line) and *Δx* = 10 nm (dash-dotted line). (**b**) Band diagram for the cell with bulk generation (*Δx* *=* 10 nm). The vertical black solid lines denote the active layer-electrode interfaces, the vertical dashed line shows the interlayer interface, the solid lines are lowest unoccupied molecular orbital (LUMO) and highest occupied molecular orbital (HOMO) energies, the dash-dotted/dotted line is the electron/hole quasi-Fermi level. The Fermi levels of electrodes are denoted by horizontal black solid lines. The vertical black dot lines denote the boundaries of the generation region. (**c**) Recombination rate of free charges *R* *=* *α(np–n*_*int*_^2^) in the bulk of the active layer; *α* is the Langevin recombination constant^25^, and *n*_*int*_ is the intrinsic concentration of charge carriers^24^. Plots (**b**) and (**c**) are given for the maximum power point.

First, we investigated how substitution of interface generation of free charges in the high-*FF* OSC for their bulk generation affects its *J–V* characteristics (*interface→bulk charge generation in OSC*). We replaced the interface generation of free charges in the OSC with bulk generation in a part of the active layer near the interface, *Δx* (see the details in SI). Figure 2 compares the results for OSC with the interface and bulk generation of free charges: panel (a) demonstrates *J–V* characteristics, panel (b) shows the band diagrams, and panel (c) displays the recombination rate of free charges. As Fig. 2a shows, the shapes of the *J–V* curves for the bulk charge generation are significantly different from that for the interfacial generation. The substitution of the interface generation for the bulk one leads to a strong drop of *FF* below the SQL (see Fig. 1b, points 5 and 6).We suggest that *FF* is low due to high recombination (Fig. 2c) of charge carriers within and near their generation region (Fig. 2b), this is discussed in details in SI.

In a symmetric numerical experiment (*bulk→interface charge generation in ISC*), we take the inorganic *p–n* junction solar cell and change charge generation in it from bulk to surface one at the interface between *p* and *n*-layers. As a result, the *FF* does not increase and remains to be equal to the SQL; moreover, the *J–V* characteristic does not change. This is expected, as the only loss mechanism in the inorganic solar cell is radiative recombination that is the same in the cells with bulk and interface charge generation. Thus, we conclude that the type of free charge generation (interface or bulk) is not a key parameter that can result in a *FF* exceeding the SQL.

### Junction type: homojunction vs heterojunction

**Figure 3:**
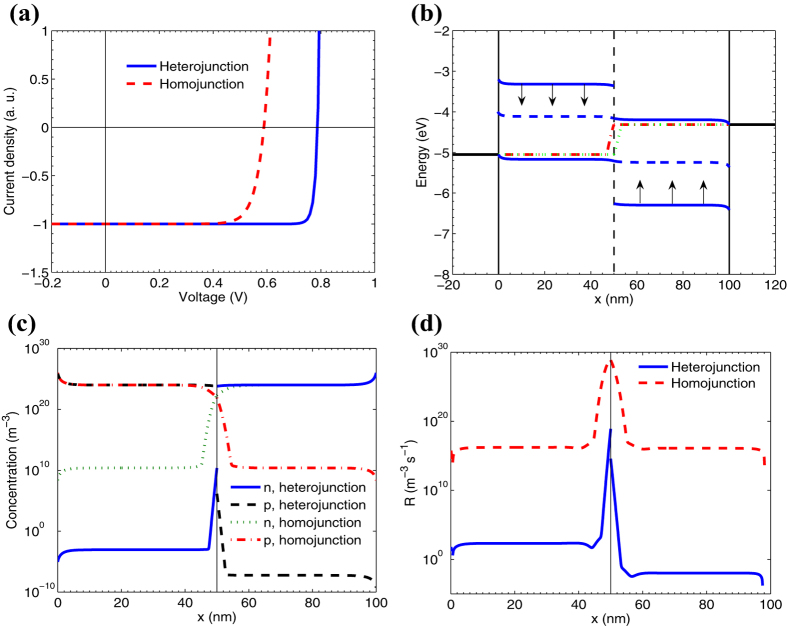
Hetero- vs homojunction. (**a**) *J–V* characteristics for the organic heterojunction solar cell (solid line) and for the corresponding hypothetic homojunction cell (dashed line). (**b**) Band diagrams for the heterojunction (solid lines) and homojunction (dashed lines) cells, respectively. Vertical arrows indicate the offset of the energy levels in the transition from hetero- to homojunction. Distribution of free electrons (*n*) and holes (*p*) concentrations (**c**) and the recombination rate *R* *=* *α(np–n*_*int*_^2^) (**d**) in the active layer for the hetero- and homojunction. The data in panels (**b**–**d**) are calculated in the maximum power point. For other details see the caption to Fig. 2.

Consider whether the junction type, i.e., hetero- or homojunction, could be responsible for *FF* exceeding the SQL. We start from the high-*FF* OSC and substitute in it the heterojunction for a homojunction (*heterojunction→homojunction in OSC*). We shift the donor lowest unoccupied molecular orbital (LUMO) and acceptor highest occupied molecular orbital (HOMO) energies of the OSC so that the heterojunction transformed into a hypothetic homojunction with a band gap of 1.05 eV and an electron affinity of 4.0 eV, with the other parameters unchanged. We will refer the donor and acceptor layers in the homojunction cell as *p*-layer and *n*-layer, respectively. Figure 3 compares the key characteristics of the hetero- and homojunction solar cells: *J–V* characteristics (a), band diagrams (b), concentrations of free electrons and holes (c) and recombination rates (d). The transformation of hetero- to homojunction decreases both *V*_*OC*_ and *FF*. The *FF* of the homojunction cell is given in Fig. 1b as a square (point 7), and it is significantly lower than the SQL. The reason for this is similar to the previous case (*interface→bulk charge generation in OSC*) — increase of the charge recombination rate in the bulk of the active layer (as shown in Fig. 3d) due to increased concentrations of minority charge carriers, i.e. electrons in *p*-layer and holes in *n*-layer (Fig. 3с). High concentrations of minority carriers appear due to lowered energy barriers at the interface in the homojunction OSC (Fig. 3b). The other details are described in SI.

In the corresponding symmetric numerical experiment, we transform the homojunction to a heterojunction (*homojunction→heterojunction in ISC*) by shifting the energy levels in the *p–n* junction solar cell model: the conducting band edge *E*_*c*_ of the *p*-layer is shifted up, and the valence band edge *E*_*v*_ is shifted down. As in the above numerical experiment related to the free charge generation type, the *J–V* characteristics are unchanged, and the *FF* remains within the SQL. Therefore, the junction type is not a key parameter responsible for *FF* exceeding the SQL.

Note that from the comparison of homo- and heterojunction in OSC and ISC one can show that the band gap also is not a parameter that is responsible for *FF* exceeding the SQL.

Using the same approach, we have tested the effect of charge mobility and dielectric constants on *FF*. In the OSC/ISC, we have substituted the charge mobilities (dielectric constants) for those of the ISC/OSC (Table 1). As a result, the *FF* decreases below the SQL in the OSC, but it remains within the SQL in the ISC. Therefore, neither the charge mobilities nor the dielectric constants are responsible for *FF* exceeding the SQL.

### Field-dependent charge recombination

In OSC, light-induced charge generation is considered as a multistep process. Excitons formed after photon absorption diffuse to the donor-acceptor interface and dissociate there into bound e/h-pairs with the electrons in the acceptor phase and the holes in the donor phase. Then these bound e/h-pairs can either recombine monomolecularly with the rate *k*_*f*_ or dissociate into free charge carriers with the rate *k*_*diss*_, which increases with the electric field at the interface *E*_*i*_. The free charges can move to the electrodes or can diffuse back to the interface and form bound e/h-pairs. If *k*_*diss*_*(E*_*i*_) ≫ *k*_*f*_ , the free charge generation rate is proportional to *k*_*diss*_(*E*_*i*_)/(*k*_*f*_ + *k*_*diss*_(*E*_*i*_)) ~1 and hence weakly depends on *E*_*i*_. However, the recombination rate of free charges *R* depends on the *E*_*i*_ as *R* ~ *k*_*f*_/*k*_*diss*_(*E*_*i*_), so it decreases with increasing the *E*_*i*_, which is almost proportional to the difference between the applied voltage *V* and the built-in voltage *V*_*BI*_. Therefore, the photocurrent *J*_*ph*_ (see eq. (1)) in OSC increases as the *V* decreases from the *V*_*OC*_down to zero (short circuit condition). This is in contrast with ISC, where the *J*_*ph*_ does not depend on the voltage as photon absorption directly generates free charges and recombination is independent of the electric field. Accordingly, in OSC, the *J–V* curve will be steeper and the *FF* can be higher than that given by equation (1). Consequently, we suggest that the key reason for unexpectedly high *FF* in OSC is field-dependent charge recombination at the donor-acceptor interface.

**Figure 4:**
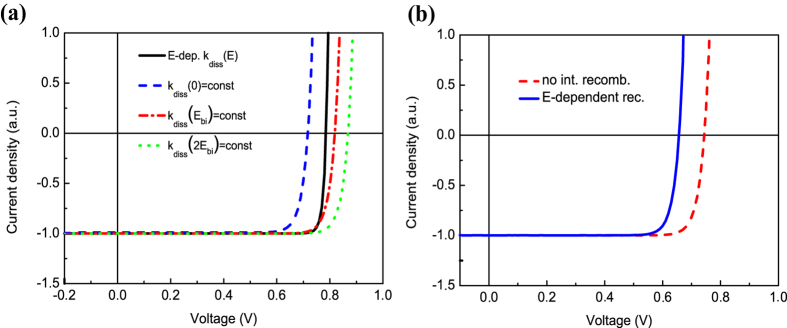
Field-dependent vs field-independent interface recombination. *J–V* characteristics for the OSC (**a**) and ISC (**b**) models with field-dependent (solid curves) and field-independent (broken curves) recombination rates at the interface between the donor/acceptor and p/n layers, respectively.

To substantiate our suggestion, we used the OSC model and made there the interface recombination field-independent (with other parameters unchanged): the bound e/h-pair dissociation rate *k*_*diss*_*(E*_*i*_) was replaced by the constants *k*_*diss*_*(0)*, *k*_*diss*_*(E*_*BI*_) or *k*_*diss*_*(2E*_*BI*_), where *E*_*BI*_ *=* *V*_*BI*_*/(L*_*1*_*+L*_*2*_) *=* *const,* where *L*_*1*_ and *L*_*2*_ are the donor and acceptor layer thicknesses, correspondingly. As a result, the *FF* decreased to the SQL as shown in Fig. 1b by points 8–10. Figure 4a compares *J–V* characteristics of the OSC with field-dependent and field-independent recombination for the three different constant rates *k*_*diss*_. The *J–V* curves for the field-independent *k*_*diss*_ are less steep than that for the field-dependent *k*_*diss*_. As a result, the *FF* for the constant *k*_*diss*_ is equal to or just below the SQL. However, for the field-dependent *k*_*diss*_, the *FF* is 91.7%, which exceeds by 7% the SQL at *V*_*OC*_ = 0.785 V. In the corresponding symmetric experiment, we took the ISC model and introduced there the field-dependent interface recombination from the OSC model with the other parameters unchanged. Figure 4b compares *J–V* characteristics for the ISC model with and without the field–dependent interface recombination. For the former, the *V*_*OC*_ significantly drops, but the *FF* slightly exceeds the SQL as shown in Fig. 1b by point 12. Without the interface recombination, the *FF* is equal to the SQL (85.3% at *V*_*OC*_ = 0.744 V, point 11 in Fig. 1b) and, with the field-dependent recombination, the *FF* reaches 84.7% exceeding by 1% the SQL at *V*_*OC*_ = 0.656 V. Therefore, using both the OSC and ISC models, we have found that the interface field-dependent recombination can result in *FF* exceeding the SQL.

### The form of k_diss_(E_i_) dependence

The field dependence of the interface recombination rate is determined by the bound e/h-pair dissociation rate *k*_*diss*_, which depends on the *E*_*i*_. The above results were obtained for the specific dependence *k*_*diss*_(*E*_*i*_) from Ref.^20^ describing electron thermoemission from a Coulumbic potential well under the electric field *E*_*i*_:
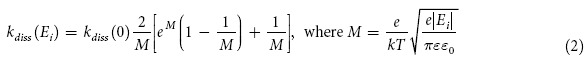


The parameter *M* is the ratio of the energy, by which the thermoemission barrier is lowered due to the electric field, to the thermal energy.

Obviously, this function, *k*_*diss*_(*E*_*i*_), is not universal and depends on the model. To reveal how the *FF* in OSC is sensitive to the field dependence of *k*_*diss*_(*E*_*i*_), we modeled *J–V* characteristics for smoother and steeper field dependences than that given by equation (2). As a smoother *k*_*diss*_(*E*_*i*_), we used the linear function of *E*_*i*_


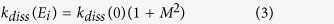


As a steeper *k*_*diss*_(*E*_*i*_), we used the exponential-square-root function of *E*_*i*_


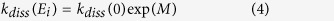


**Figure 5:**
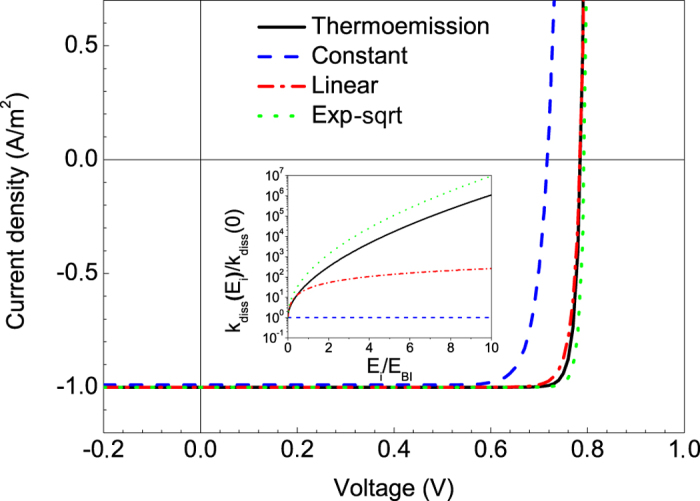
*J–V* characteristics of OSC with different forms of bound e/h-pair dissociation rate field dependences *k*_*diss*_*(E*_*i*_). Inset displays the *k*_*diss*_*(E*_*i*_) dependences described by equations (2, 3, 4).

Figure 5 shows *J–V* characteristics for OSC with these three different dependences *k*_*diss*_*(E*_*i*_) and for the constant *k*_*diss*_(*E*_*i*_) = *k*_*diss*_(0). The corresponding *FF*s are plotted in Fig. 1b by triangles (points 1, 8, 13 and 14). For the cell with the linear *k*_*diss*_(*E*_*i*_) (Eq. (3)) the *FF* is 89.2% that is higher by 4% than the SQL at *V*_*OC*_ = 0.784 V. For the cell with the exponential-square-root *k*_*diss*_(*E*_*i*_) (Eq. (4)) the *FF* is 92.9% that is higher by 8% than the SQL limit at *V*_*OC*_ = 0.793 V. Therefore, our calculations show that for OSC with *FF* exceeding the SQL the *E*_*i*_-dependence of *k*_*diss*_(*E*_*i*_) must be an increasing function, and the form of this dependence is not essential. Nevertheless, the steeper the function *k*_*diss*_(*E*_*i*_), the steeper the corresponding *J–V* curve near *V*_*OC*_, and the higher *FF* can be reached.

## Discussion

Using numerical drift-diffusion modeling, we have shown that the *FF* in a bilayer OSC can be higher than the SQL for *FF* established for ISC. The OSC model with various numerical parameters (charge mobilities, band gaps, doping level) can result in *J–V* curves with the *FF* higher than the SQL for a given *V*_*OC*_ (see Fig. 1b). Confronting the drift-diffusion models of OSC (planar heterojunction) and ISC (*p–n* junction), we have identified the reason responsible for high *FF* in OSC. Such OSC properties as heterojunction and interface charge generation facilitate a high *FF* in OSC as compared to the homojunction and bulk charge generation in ISC because of lower charge recombination; however, they cannot provide the *FF* beyond the SQL. Such a high *FF* is due to interface charge recombination that must decrease with increasing the electric field at the heterojunction interface. Indeed, on the one hand, the *FF* drops lower than the SQL if we use an OSC model similar to a *p–n* junction cell by making the recombination of free charges field-independent. On the other hand, the *FF* increases above the SQL if we introduce field-dependent interface recombination in the *p–n* homojunction model. Thereby we claim that the *FF* in OSC can exceed the theoretical limit for inorganic *p–n* junction solar cells, and this is due to field-dependent recombination of free charges at the donor-acceptor interface. The rate of such field-dependent recombination should be much lower than the free charge generation rate, in other words the dissociation rate of bound e/h-pairs *k*_*diss*_(0) should be much higher than monomolecular recombination rate of bound e/h-pairs *k*_*f*_. If this condition is not satisfied, the recombination will decrease the *FF* in organic solar cells^19^. To summarize, field-dependent recombination is the necessary but not sufficient condition for *FF* exceeding SQL.

Note that the model used does not account for light absorption, exciton diffusion, and formation of bound electron-hole pairs at the donor-acceptor interface. All these processes determine the generation rate of bound electron-hole pairs *G*_*S*_, which is an input parameter in the model, and we put it constant in our numerical studies; moreover, we normalized all the *J–V* characteristics to *G*_*S*_. In addition, when we changed the heterojunction for the homojunction, the donor and acceptor band gaps narrowed, and the donor LUMO – acceptor LUMO energy difference reduced. The former increases the number of absorbed photons, and the latter decreases the driving force that splits excitons. Evidently, this will influence *G*_*S*_ and the device efficiency. However, we put *G*_*S*_ constant in our numerical studies as our aim was to show how the differences in free charge generation, recombination, and transport in organic and inorganic solar cells can lead to an unexpectedly high *FF* in the former.

In this study, we assumed the presence of a sharp and flat interface between the donor and acceptor layers. Note that a two layer organic structure, e.g., P3HT/PCBM, is susceptible to intermixing^29^. However, intermixing at the donor-acceptor interface could be suppressed by using cross-linked donor or/and acceptor layers^30^,^31^. Essentially, the results obtained in our work for bilayer OSC can be naturally extrapolated to ordered bulk heterojunction OSC, i.e., those with large-area non-planar heterojunction interface where the donor (acceptor) phase is in contact with anode (cathode) and not with cathode (anode).

The maximal experimental values of *FF* achieved in OSC are high (about 80%) but still lower than the SQL (the spheres in Fig. 1b and the last two rows in Table 2). This is because in real OSC it is rather difficult to achieve the conditions, which can provide the ultimate *FF*. The model used does not take into account a number of effects that can limit the *FF* in real devices such as disorder of energy states in organic semiconductors, defect- and contact-mediated recombination, etc. However, such effects can be incorporated in the model and at least in principle can be strongly suppressed. Importantly, the ultimate efficiency of OSC is obviously below the SQ efficiency limit for a single-junction cell because of an extra energy loss for exciton dissociation. This energy loss generally decreases the *V*_*OC*_; however, the *FF* is already not limited by the SQ model. As a result, the *J–V* curves can be steeper than that in the SQ model but with lower *V*_*OC*_.

In conclusion, drift-diffusion modeling shows that the *FF* in OSC can exceed the SQL for *FF* established for *p–n* junction ISC. We conclude that the field-dependent recombination of free charges at the donor-acceptor interface is responsible for the *FF* beyond the SQL. This recombination rate must drop with increasing the electric field at the donor-acceptor interface, and the dissociation rate of bound e/h-pairs should be much higher than the rate of their monomolecular recombination; the form of the recombination rate dependence on the electric field is not crucial, but the steeper it, the higher *FF* can be reached. On the one hand, field dependence of the charge recombination rate is the necessary condition for *FF* exceeding SQL but it is not sufficient. On the other hand, heterojunction and interface generation in OSC facilitate reaching high *FF* but are not necessary conditions for *FF* exceeding SQL. Our findings highlight an unnoticed advantage of OSC and can be used in their further improvement.

## Methods

### Drift-diffusion *p–n* junction model

The *p–n* junction model for ISC is based on the same equations used in the bilayer OSC model^24^: Poisson equation, current-continuity equations for electrons and holes, and drift-diffusion equations for electron and hole currents. The current-continuity equations are:


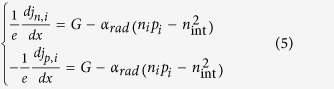


where *j*_*n,i*_ and *j*_*p,i*_ are current densities of electrons and holes, *G* is the charge generation rate, *α*_*rad*_ is the bimolecular radiation recombination rate, *n*_*i*_ and *p*_*i*_ are the electron and hole concentrations, index *i* = 1 and 2 corresponds to the *p* and *n*-layer respectively.

As the surface generation and recombination at the interface between *p*- and *n*-type layers are absent, the matching conditions for the electron and hole current densities are:


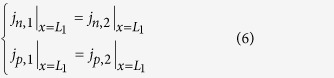


where *L*_*1*_ and *L*_*2*_ are the *p*- and *n*-layer thicknesses, correspondingly. In the *p–n* junction model, the numerical value of bulk generation rate *G* is taken such that the total quantity of absorbed photons equals to the quantity of photogenerated bound electron-hole pairs at the donor-acceptor interface in the OSC model:


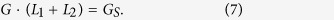


Another difference between the bilayer OSC and *p–n* junction models is in the boundary conditions. In the latter, we suggest ideal contacts, i.e., membranes that are permeable for the majority charge carriers and block the minority charge carriers^32^. Therefore, the current densities of the latter at the contacts with electrodes are equal to zero:


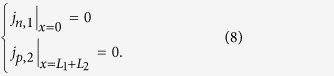


## Additional Information

**How to cite this article**: Trukhanov, V.A. *et al.* Fill factor in organic solar cells can exceed the Shockley-Queisser limit. *Sci. Rep.* **5**, 11478; doi: 10.1038/srep11478 (2015).

## Supplementary Material

Supplementary Information

## Figures and Tables

**Table 1:** Input parameters of bilayer organic and inorganic *p–n* junction solar cells; for the planar heterojunction, the parameters of donor and acceptor layers are divided by slash marks.

Process/parameter	Organic solar cell planar heterojunction	Inorganic solar cell *p–n* junction
Generation of free charges	interface	bulk
Junction type	heterojunction	homojunction
Interface recombination	field-dependent	none
Bulk recombination	Langevin	radiative, *α*_*rad*_ = 3 × 10^−18^ m^3^/s
Band gap, *E*_*g*_	1.85/2.1 eV	1.12 eV
Electron (hole) mobilities, *μ*_*n*_ *(μ*_*p*_)	10^−10^ (10^−7^)/10^−7^ (10^−10^) m^2^/(V s)	0.12 (0.03) m^2^/(V s)
Dielectric constant, *ε*	3/4	12
Doping level, *N*_*d*_	10^24^ m^−3^	10^24^ m^−3^

**Table 2:** Open-circuit voltages (*V*_*OC*_) and fill factors (*FF*) of modeled (rows 1–14) and experimental (the last two rows)^27,28^ solar cells.

Point number	Сell type	Description	*V*_*OC*_ (V)	*FF* (%)	SQL for *FF* (%)
1	Organic	Non-modified (doped)	0.785	91.6	85.8
2	Organic	*N*_*d*_ = 0, *μ*_*n*_ = *μ*_*p*_ = 10^−5^ m^2^/(V s)	0.595	87.1	82,6
3	Organic	*N*_*d*_ = 0, thin active layers *L*_*1,2*_ = 30 nm	0.694	87.4	84.5
4	Organic	*N*_*d*_ = 0, LUMO(A)-HOMO(D) = 0,6 V	0.292	73.7	71.6
5	Organic	Homojunction	0.587	79.3	82.4
6	Organic	Bulk generation, *Δx* = 1 nm	0.765	48.7	85.6
7	Organic	Bulk generation, *Δx* = 10 nm	0.739	20.5	85.2
8	Organic	*k*_*diss*_(*E*) = *k*_*diss*_(0) = *const*	0.716	84.8	84.8
9	Organic	*k*_*diss*_(*E*) = *k*_*diss*_(*E*_*BI*_) = *const*	0.819	86.2	86.3
10	Organic	*k*_*diss*_(*E*) = *k*_*diss*_(2*E*_*BI*_) = *const*	0.868	86.6	86.9
11	Inorganic	Non-modified	0.744	85.3	85.3
12	Inorganic	Field-dependent interface recombination	0.656	84.7	83.8
13	Organic	Linear *k*_*diss*_(*E*)	0.784	89.2	85.8
14	Organic	Exponential square-root *k*_*diss*_(*E*)	0.793	92.9	85.9
−	Organic	Bulk heterojunction PTPD3T:PCBM^27^	0.795	79.6	86.0
−	Organic	Planar heterojunction DIP/C_60_^28^	0.930	74.3	87.5

The last column shows the *FF* according to the Shockley-Queisser limit (SQL). All the data are also presented in Fig. 1b.
