# Supplementary material for: Fill factor in organic solar cells can exceed the Shockley-Queisser limit
Source: Sci Rep. 2015 Jun 22;5:11478. doi: 10.1038/srep11478 (PMC4476110; doi:10.1038/srep11478)
Supplement: Supplementary Information [file srep11478-s1.doc]

**Supplementary Information: Fill factor in organic solar cells can exceed the Shockley-Queisser limit**

V.A. Trukhanov, V.V. Bruevich, D.Yu. Paraschuk*

Faculty of Physics and International Laser Center, M.V. Lomonosov Moscow State University, Moscow, 119991, Russia

* Correspondence to e-mail: paraschuk@gmail.com

**Supplementary Figure S1 |** Band diagrams for the heterojunction (a) and homojunction (b) cells at the maximum power point *V_MP_*=0.736 and 0.467 V, respectively. For other details see the caption to Fig. 2 of the main text.

**Supplementary note 1: Interface→bulk charge generation in OSC.**

We replaced the interface generation of free charges in the bilayer OSC with bulk generation in a part of the active layer near the interface, *Δx*, i.e., pairs of free carriers were generated at *x* є (*L*_1_-*Δx*, *L_1_*+*Δx*), where *L_1_* is the donor layer thickness (*x*=*L_1_* is a coordinate of the donor-acceptor interface), and the interface generation rate of electron-hole pairs *G_S_*=0. The bulk generation rate of free electron-hole pairs, *G*, was such that the total amount of generated carriers was the same as for the cell with interface generation:

$$G=\left\{\begin{matrix} G_{S}/2\Delta x, x\in(L_{1}-\Delta x, L_{1}+\Delta x) \\ 0, x\notin(L_{1}-\Delta x, L_{1}+\Delta x) \end{matrix}\right. .$$

As Fig. 2a in the main text shows, the *J-V* curve for the lower *Δx* is more similar to the case of the interfacial generation while for the larger *Δx* the curve significantly changes. The substitution of the interface generation for the bulk one leads to decreased *J_SC_*, *V_OC_*, and a strong drop of *FF* from 91.7% to 49.4% for *Δx*=1 nm and to 21.0% for *Δx*=10 nm. We suggest that *FF* is low due to recombination of charge carriers within and near their generation region, 2*Δx*, because there are carriers of both types, as evidenced by the strong splitting of quasi-Fermi levels at the band diagram in Fig. 2b. The distribution of recombination rate in the bulk of the active layer with interface and bulk generation is given in Fig. 2c; the recombination rate in the cell with bulk generation in the region within *Δx* from the interface is many orders of magnitude higher than that in the cell with interface generation. This recombination leads to losses of charges and drop of *FF* below the Shockley-Queisser limit.

**Supplementary note 2: Heterojunction→homojunction in OSC.**

Figure 3 in the main text compares the key characteristics of the hetero- and homojunction solar cells. To analyze the results, consider the distribution of free charges in the active layer in the maximum power point.

As follows from the band diagram in Figure S1a, there are high energy barriers at the heterojunction interface so that the photogenerated electrons (holes) in the acceptor (donor) layer (we will refer to them as photoinduced majority carriers) cannot enter another layer, i.e., the electrons (holes) cannot enter the donor (acceptor) layer. As a result, the concentrations of the photoinduced minority carriers (Fig. 3c), i.e., holes in the acceptor layer (green dashed line at 50<*x*<100 nm) and electrons in the donor layer (blue solid line at 0<*x*<50 nm), are extremely low. Note that the photoinduced majority carriers are generated only in the corresponding layers because of exciton dissociation at the interface. In the homojuction cell, the HOMO and LUMO energy levels in both layers are aligned at the interface (Fig. S1b) so that the energy barriers associated only with band bending are smooth and lower at voltages near *V_OC_*. Therefore, after exciton dissociation, the electrons (holes) can easily diffuse back to the *p*-layer (*n*-layer) and recombine there with the corresponding photoinduced majority carriers reducing the cell performance. As follows from Fig. 3c,d, the concentrations of the photoinduced minority carriers and their recombination rate are more than ten orders higher in the homojunction as compared with the heterojunction. Importantly, these concentrations and recombination rates are particularly high near the junction interface. The increased recombination in the homojunction at voltages near *V_OC_* results in a decreased photocurrent, *FF* and *V_OC_* itself. Accordingly, *FF* in the heterojunction cell is higher than that of the homojunction cell due to the interface energy barriers, which prevent penetration of photoinduced holes (electrons) in the acceptor (donor) layer and, hence, reduce recombination losses.
